# Work of the past Quarter

**Published:** 1916-09-09

**Authors:** 


### THE LONDON HOSPITAL.

### Work of the Past Quarter.

The report of the Committee, presented to the Quarterly Court on September 6, opens with an expression of pleasure at the return of Lord Knutsford, the chairman, to his work at the hospital after his accident.

### Admission of Officers.

The chief circumstance of interest since the last Court is the fact that, at the request of the War Office, four wards for the admission of officers have been thrown open. Accommodation for a hundred cases was ready in two days' time.

The civilian medical men patients from the four wards chosen, who were seriously ill, were accommodated by putting up extra beds in other wards. It was decided that half of George Ward should be dismantled and turned into a mess or sitting-room. Mr. Oatley, the surveyor of the hospital, removed all the beds and "hospital" fittings, and in their place put carpets, chairs, tables, hot plates, etc., in the course of twenty-four hours. Thanks to the kindness of Mr. Alfred Salmon, who has great experience in such matters and has constantly helped us, we were able to obtain a staff of cooks and waitresses so that the meals should be suitably served. The total number of officers treated to date is 258.

### Employees of Military Age.

Application has been made to the local Tribunal for the exemption of many valuable employees, such as *x*-ray operators, theatre assistants, clinical laboratory assistants, dispensers, and men in skilled trades under the Works Department, so that the result of the appeal is awaited with some anxiety. A scheme has been prepared, and is now in operation, for giving allowances to all employees who have been called up for service.

### Alteration of Secretary's Title.

The secretary has been in residence since the beginning of the war, and the committee have decided to revive the old title of "house governor," under which style Mr. Morris will be known in future.

### Recommendations of the Economy Committee.

As reported to the last Quarterly Court, an Economy Committee was appointed to go into the detailed working of every department to see if any possible reduction in expenditure or the work could be made.

Their report states that the committee has not many suggestions to make which can, to any very great extent, lessen the expenditure of the hospital. This result, though largely negative, is considered gratifying, as every department examined has already reached a point beyond which little further economy can be expected. The only possibility of reducing expenditure to any very large extent must be by some entire change of system or lowering of standard.

Such recommendations as the committee make include the following:—

"That the dispensary day staff be slightly reduced by not replacing all the men as they are called up. That lady dispensers be engaged, properly qualified. That the appointment of night dispenser be abolished. That the staff be requested to restrict the use of oxygen. That serious endeavour be made to reduce the number of unnecessary new and chronic cases of out-patients. That the receiving-room during certain hours should be open for none but emergency cases. That insured patients be seen only when they bring a form filled in by the panel doctor asking for an opinion on the case. That no medicine be given to panel patients. That the payment by out-patients for treatment be increased from 3d. to 4d. That payment for treatment shall be at the minimum rate of 6d. per attendance in the light, radium, massage, electrical, bath, and dental departments. That an attempt be made to reduce the number of radiographs taken. That the attendance of Poor-Law and provincial patients be restricted. That the consumption of hot water in baths and the use of electric fans be restricted. That the use of ice be reduced if possible. That the staff be asked to keep a careful watch on the extras ordered to diets, such as bacon and eggs. That work of the dental and skin departments be reduced."

### Duty Paid on Alcohol.

The decision of the Government to give a rebate to hospitals, under certain conditions, of the duty paid on alcohol used for medicinal purposes is worth about £200 a year to the hospital.

### Help by a Member of the Committee.

Warm thanks are expressed to Mr. Foster, who since the beginning of the war has worked voluntarily and without a break in the house governor's office from 9 a.m. to 7 p.m.

### Zeppelin Raids.

By arrangements with the police, private and confidential warning is given if Zeppelins are on the coast anywhere and when they are approaching the London area. On the receipt of the second and urgent warning, arrangements are made for dealing with two possible events, the incoming of large numbers of injured from the streets, and the possibility of the hospital being struck by a bomb.

